# Antimicrobial Stewardship Programs: Appropriate Measures and Metrics to Study their Impact

**DOI:** 10.1007/s40506-014-0015-3

**Published:** 2014-04-26

**Authors:** Andrew M. Morris

**Affiliations:** 1 Department of Medicine, Mount Sinai Hospital and University Health Network, Toronto, ON Canada; 2 Faculty of Medicine, Department of Medicine, University of Toronto, Toronto, ON Canada; 3 Mount Sinai Hospital, 415-600 University Avenue, Toronto, ON M5G 1X5 Canada

**Keywords:** Antimicrobial stewardship, Metrics, Measures, Days of therapy, Length of therapy, Defined daily doses, Appropriateness, Mortality, Balancing measures, Resistance, Candidemia, *C. difficile*

## Abstract

Antimicrobial stewardship is a new field that struggles to find the right balance between meaningful and useful metrics to study the impact of antimicrobial stewardship programs (ASPs). ASP metrics primarily measure antimicrobial use, although microbiological resistance and clinical outcomes are also important measures of the impact an ASP has on a hospital and its patient population. Antimicrobial measures looking at consumption are the most commonly used measures, and are focused on defined daily doses, days of therapy, and costs, usually standardized per 1,000 patient-days. Each measure provides slightly different information, with their own upsides and downfalls. Point prevalence measurement of antimicrobial use is an increasingly used approach to understanding consumption that does not entirely rely on sophisticated electronic information systems, and is also replicable. Appropriateness measures hold appeal and promise, but have not been developed to the degree that makes them useful and widely applicable. The primary reason why antimicrobial stewardship is necessary is the growth of antimicrobial resistance. Accordingly, antimicrobial resistance is an important metric of the impact of an ASP. The most common approach to measuring resistance for ASP purposes is to report rates of common or important community- or nosocomial-acquired antimicrobial-resistant organisms, such as methicillin-resistant Staphylococcus aureus and Clostridium difficile. Such an approach is dependent on detection methods, community rates of resistance, and co-interventions, and therefore may not be the most accurate or reflective measure of antimicrobial stewardship interventions. Development of an index to reflect the net burden of resistance holds theoretical promise, but has yet to be realized. Finally, programs must consider patient outcome measures. Mortality is the most objective and reliable method, but has several drawbacks. Disease- or organism-specific mortality, or cure, are increasingly used metrics.

## Introduction

Antimicrobials are prescribed in up to a third of hospital inpatients, often inappropriately [1–3], and more than two thirds of critically ill patients are on antimicrobials at any one time [4]. Because of our current understanding of antimicrobial resistance—i.e., that it results from Darwinian selection pressure on bacteria and fungi from antimicrobials—coupled with the fact that much antimicrobial resistance coincides with increased antimicrobial consumption [5–7], efforts to try and curb the unnecessary use of antimicrobials have gathered increased attention [8, 9••, 10]. Although it is widely accepted that antimicrobial use accelerates the development of resistance, the challenge with antimicrobial prescribing lies in the need to balance two conflicting goals: the provision of therapy that is adequate to treat documented or presumed infection, and the minimization of antimicrobial use to avoid adverse drug events (e.g., *Clostridium difficile* infection and allergy) and the emergence of antimicrobial resistance, and to reduce costs.

The first use of the term “antimicrobial stewardship” probably dates back to an article in 1996, when McGowan and Gerding asserted that appropriate use of antimicrobials might avert or even reverse trends in antimicrobial resistance [11]. Since that time, antimicrobial stewardship programs (ASPs) have continued to become more prevalent in tertiary and quaternary care hospitals, although smaller, community-based hospitals are increasingly paying attention to antimicrobial use. However, hospitals and their ASPs are also struggling with identifying appropriate measures of success for such programs. This article reviews the various measures that are available to ASPs and administrators who oversee such programs, to help guide antimicrobial stewards with assessment of their programs and interventions.

## Antimicrobial measures

Consumption measures refer to metrics that reflect an aggregate or average amount of antimicrobials being consumed at the level of the patient, a hospital unit or service, or an entire institution. They are often reported with patient-day as the denominator (i.e., per 1,000 patient-days) to standardize according to bed utilization, although mean daily dose and total quantity of therapy have also been reported [12••]. ASPs will use any of several different sources for this information, including ordered antimicrobials, dispensed antimicrobials, and administered antimicrobials. The most accurate and relevant of these measures is administered drug [13•], but most institutions do not have the infrastructure or capability to assess this measure. Some other hospitals choose to use purchased antimicrobials, obtaining business data rather than clinical data. The downsides of this approach are obvious, but imperfect yet consistently acquired data is likely better than no data at all.

### Antimicrobial defined daily dose

The most widely accepted measure is defined daily dose (DDD), a metric that was developed in the 1970s and has been further refined and promoted by the World Health Organization Collaborating Centre for Drug Statistics Methodology. DDD “is the assumed average maintenance dose per day for a drug used for its main indication in adults” [14]. In simple terms, a DDD is the amount of drug that a typical patient might receive on any day for therapeutic purposes. DDD was never intended to be used as a metric for antimicrobial stewardship. There are particular reasons why it probably should *not* be used as a metric to study the impact of antimicrobial stewardship. For one, DDD biases against combination therapy, even when that therapy might be narrower spectrum (e.g., combining metronidazole and cefazolin for an intra-abdominal infection will result in double the DDDs of using piperacillin–tazobactam or meropenem). Because DDDs assume routine dosing, programs might be “penalized” for using appropriately higher doses when necessary, such as in patients with obesity or who have central nervous system infections. Conversely, reduced dosing of antimicrobials for renal dysfunction will underestimate antimicrobial exposure [15]. Similarly, using DDDs for pediatrics results in rather uninterpretable data. Finally, because administered dosing often differs from the DDD dosing standard for several drugs, it is difficult to infer days of therapy (DOT) from DDDs or to make conclusions about the relative use of one antimicrobial compared with another [16]. However, an important advantage of using DDDs is the relative ease of hospital systems to report consumption using DDDs: most pharmacy departments have a mechanism to calculate overall prescription, dispensing, or consumption of a quantity of antimicrobials, allowing DDDs/1,000 patient-days to be relatively easy to calculate if bed utilization is also available. Additionally, institution-wide consumption can be benchmarked against similar institutions. The landmark guidelines on antimicrobial stewardship by the Infectious Diseases Society of America and Society for Healthcare Epidemiology of America advocated for DDDs/1,000 patient-days as a universal metric for hospital-based ASPs [10].

### Antimicrobial days of therapy

In contrast to DDD, DOT offers more clinical relevance to the healthcare provider [17]. Whereas DDDs hold a dubious relationship with the treatment patients actually receive, DOTs do not. They are relatively intuitive, and correlate with evidence from clinical trials where antimicrobials are given for a fixed duration. Some scenarios do pose problems, however (e.g., drugs with a long half-life, especially in the setting of renal failure). This has led to the concept of “exposure days” rather than DOT. However, it appears that the distinction is not relevant from a practical point of view [18]. Most importantly, most hospitals—especially smaller ones—are unable to calculate DOTs accurately and easily. A limitation that DOT shares with DDD as a metric of antimicrobial stewardship impact is the incentive to use broad-spectrum monotherapy (because a patient receiving two antimicrobials for 7 days contributes 14 DOTs). As mentioned above, DOT is broadly applicable to a pediatric population (including neonates), whereas most other measures (including DDD) are not.

### Antimicrobial-free days

A measure that holds particular appeal is antimicrobial-free days. This avoids (or overcomes, depending on perspective) the issues related to spectrum of therapy, and monotherapy versus duotherapy, and focuses on whether patients are receiving antimicrobials or not. It has been mostly used as a disease-specific consumption measure (specifically ventilator-associated pneumonia) [19, 20], but it also holds appeal as a broader measure of ASP impact.

### Grams of antimicrobial therapy

Using a different approach to address the problems with DDDs, some investigators have become interested in the overall mass of antimicrobials being consumed by patients [13•]. It is unclear what this offers above and beyond DOT or DDD. Additionally, it is unclear if mass of antimicrobial administered has a better relationship to antimicrobial resistance and other adverse effects from antimicrobial use than other commonly used metrics.

### Antimicrobial cost of therapy

Perhaps the easiest metric for most hospitals to acquire is cost of therapy (COT) [21]. Many providers are reluctant to report cost because (in most healthcare jurisdictions) antimicrobial acquisition costs are variable from institution to institution, and also vary over time. Additionally, they are markedly affected by genericization of antimicrobials. However, because newer drugs tend to be more broad spectrum and expensive, COT is one of the few metrics readily available that address antimicrobial spectrum of therapy in some manner. Additionally, because cost savings are an important outcome for ASPs, reductions in COT are useful for ASPs to report [22–25, 26••, 27]. Unfortunately, COT should not be used for benchmarking purposes because of the differences in purchasing agreements between institutions, and variability in costs from country to country.

### Antimicrobial prevalence

Because relatively sophisticated data systems are required to measure the various indicators listed above, there is a need in some healthcare settings to more nimbly measure consumption in a reliable manner. One such method is point prevalence. One of the early studies to perform this was the European Prevalence of Infection in Intensive Care (EPIC) study, later replicated in EPIC II, which was an international study of infection in intensive care [4, 28]. Increasingly, larger coordinated efforts are using point prevalence reports of antimicrobial exposure as a replicable measure of antimicrobial consumption in hospitals [29•, 30, 31]. Malcolm et al. have reported that point prevalence studies can successfully drive quality improvement in antimicrobial prescribing [29•].

### Effect of length of stay on aggregate consumption measures

Care in acute care hospitals globally has evolved, resulting in a higher acuity of illness for inpatients, coupled with increasingly short lengths of hospital stay. This has resulted in patients who are admitted with infection remaining on antimicrobials until (and often after) discharge. This has the potential effect of increasing the aggregate antimicrobial consumption for inpatients even if antimicrobial practice hasn’t changed substantially [32••]. Additionally, because the course of therapy is censored at the time of discharge, it is often unclear what total duration of therapy patients have received.

### Disease-specific consumption measures

Aggregate data are the most commonly used metrics for ASPs because they are the most readily accessible. However, their greatest limitation relates to the fact that they do not adequately account for clinical indications: reports using DDD or DOT do not correct for the prevalence of infection in a population. Accordingly, ASPs are increasingly interested in looking at antimicrobial consumption for specific conditions [e.g., community-acquired pneumonia (CAP) or methicillin-resistant *Staphylococcus aureus* (MRSA) bacteremia], using either COT or DOT. Perhaps the most appropriate measure when looking at disease-specific therapy is duration of therapy or length of therapy (LOT) [33–36]. LOT differs from DOT in that the number of antimicrobials is largely irrelevant. Additionally, it accounts for dosing intervals beyond 1 day (e.g., patients receiving vancomycin every 48 h). Finally, LOT accounts for best practice by not penalizing programs for changing antimicrobials once susceptibilities are known. This is most important when programs look at ordered or dispensed drugs, rather than administered drugs. Antimicrobial-free days, mentioned previously, are inversely related to LOT.

### Targeted antimicrobials

Some antimicrobials attract more attention than others. There are many potential reasons for this: they may be more expensive, more broad spectrum, more toxic, have a stronger association with *C. difficile*, or resistance to them may be a greater problem than it is with other agents. Vancomycin and anti-pseudomonal (and other broad-spectrum) antimicrobials are commonly selected agents [37–46]. There is sufficient evidence that ASPs can achieve reductions in targeted antimicrobial use. However, it is not entirely clear that such approaches reduce overall antimicrobial use or unnecessary antimicrobial use (i.e., using an antimicrobial when none is needed). It also does not address whether an agent with an appropriate spectrum of activity is prescribed. This approach lends itself to “squeezing the balloon,” shifting antimicrobial selection pressure to cheaper agents [47].

### Appropriateness measures

Perhaps the most desired measure of antimicrobial use to determine whether an ASP is effective is antimicrobial “appropriateness.” When assessed comprehensively, this would theoretically state whether the right agent, with the appropriate antimicrobial activity to treat the infection of concern, is being provided at the right dose, route and schedule, for the right duration, accounting for allergies, drug interactions, and potential toxicities. In some ways, this is the ideal metric for consumption. Unfortunately, it appears impossible to measure in a reliable, valid, and widely accepted manner. Some authors have suggested concordance with treatment guidelines [43, 48, 49]. However, this assumes that antimicrobial guidelines are a gold standard of best practices. In addition to most guidelines having been developed without a particular eye on antimicrobial stewardship principles, many guidelines lack the quality and development rigor that validate them as a measure of appropriateness [50, 51]. Further, assessing appropriateness is labor-intensive; measuring it using point-prevalence methodology (e.g., assessing appropriateness of therapy on a particular day throughout a ward or institution) is a way of making measures of appropriateness more feasible.

### Process measures

Although “appropriateness” could be considered a process measure, most process measures in antimicrobial stewardship relate to the various factors that should lead to better prescribing. These can include (but are not limited to) documenting an indication for the antimicrobial, filling in an antimicrobial order form, or mandating an infectious diseases consult [38, 52]. As with all process measures, they may be important to ensure that a quality improvement methodology is doing as it is intended, but they do not truly reflect the quality of antimicrobial prescribing, or the effect of the prescribing on important outcomes.

## Microbial measures

At the time of writing, there are no prospective randomized studies demonstrating that antimicrobial stewardship can reduce antimicrobial resistance, even though there is a plethora of observational studies suggesting that improving antimicrobial use can improve antimicrobial resistance. Because reduced resistance is one of the primary justifications for ASPs, being able to accurately and reliably report on antimicrobial resistance is an important metric.

### Antimicrobial resistance prevalence

#### Selected antimicrobial-resistant organisms

The initial institutional response to antimicrobial resistance by healthcare was, primarily, using principles of infection prevention and control. Patients were screened for common and/or worrisome antimicrobial-resistant organisms (AROs) such as MRSA and vancomycin-resistant enterococci. Accordingly, hospitals in some jurisdictions were expected to report cases or rates of hospital-acquired infection due to these AROs, with the understanding that new cases of these (primarily nosocomial) organisms represented a failure of infection prevention and control practices. Further, for organisms such as MRSA, the first wave of cases were due to (what eventually became referred to as) hospital-acquired MRSA (HA-MRSA) beginning in the 1960s [53]. Unfortunately, relatively little attention was paid to antimicrobial use as a driving factor for HA-MRSA. With the emergence of community-acquired MRSA (CA-MRSA), approaches to screening, diagnosis, and management of MRSA changed. Further, institutions continued to ignore any relationship between antimicrobial stewardship and MRSA, with the understanding that the epidemiology of CA-MRSA could not be altered by changes in antimicrobial use. Ironically, most guidance on responding to the emergence of CA-MRSA focused on using more, broader-spectrum antimicrobials [54]. The extreme of this example is correlating the incidence of *C. difficile* infection with antimicrobial use [42, 43, 45, 55–60]. Usually this is reported in juxtaposition with a cluster or outbreak of *C. difficile* infection.

Despite these trends, some authors have advocated for using prevalence of AROs as an antimicrobial stewardship metric [17, 61]. It seems unlikely, though, that antimicrobial use will, alone, have a significant impact on hospital prevalence of community-acquired organisms such as CA-MRSA. Specifically, community prevalence of AROs is far more likely to modify hospital prevalence than antimicrobial stewardship measures. Further, because screening approaches might influence “prevalence,” heterogeneity might exist due to differences in screening strategies rather than in real differences between populations.

McGowan has discussed the need to identify microbial benefits to antimicrobial stewardship in detail in his excellent review [26••]. He points out that there is a surprising paucity of literature on the beneficial effects of antimicrobial stewardship on antimicrobial resistance. However, in a recent Cochrane Review (an update to an earlier review in 2005), Davey and colleagues were able to show that there is increasing evidence that antimicrobial stewardship interventions can influence antimicrobial resistance [62••]. In particular, interventions intended to decrease excessive prescribing were associated with a reduction in *C. difficile* infections and colonization or infection with aminoglycoside- or cephalosporin-resistant Gram-negative bacteria, MRSA, and vancomycin-resistant *Enterococcus faecalis* [62••]. The problem in looking at resistance in a single or few species is the potential for “squeezing the balloon” [47]. Specifically, ASPs may assess their impact by reporting on the reduction of resistance to a certain (measured) class of antimicrobial when, in fact, resistance to another antimicrobial or class of antimicrobials increases because of shifted prescribing patterns.

#### Antibiograms

Recognizing that tracking selected AROs—and trying to influence their prevalence—is unlikely to be clearly related to antimicrobial use, a more holistic approach to antimicrobial resistance might be exploring overall antimicrobial resistance from hospitalized patients. The representation of susceptibilities of multiple organisms to multiple agents is called an antibiogram. Unfortunately, there are no proposed aggregate measures in the literature that offer guidance to resistance for multiple antimicrobials in multiple organisms [63•]. The closest approximation to this is the Drug Resistance Index (DRI) proposed by Laxminarayan and Klugman [64]. However, the DRI is a measure that was conceived for larger populations, modeling it on the consumer price index to reflect community based antimicrobial consumption and resistance. Although it is conceivable (and likely) that antimicrobial use (and stewardship) will have an impact on antimicrobial susceptibilities in-hospital, there are very little data to show this in a broad manner.

## Clinical outcomes

Perhaps surprisingly, clinical outcomes have been reported in a minority of studies evaluating antimicrobial stewardship interventions [26••]. The reasons are unclear, but likely relate to the data sources for most ASP interventions (administrative and pharmacy databases), in addition to a biased belief that “better” antimicrobial use must be better for the patient. At a minimum, clinical outcomes are useful balancing measures, to help ensure that patients are not harmed through efforts to better rationalize antimicrobial prescribing.

### Mortality

Perhaps the most objective clinical outcome for ASPs is mortality. There are obvious problems with using mortality as an outcome, especially when most ASPs will evaluate their interventions as before–after studies, rather than randomized controlled interventions. In particular, secular trends in healthcare mortality may result in lower “after” mortality (because of concurrent quality improvement initiatives) or higher “after” mortality (because of a trend to limit hospitalization to sicker and/or older patients). Other factors might affect mortality, as well. Accordingly, mortality results should be interpreted with caution. Further, it is unclear if most antimicrobial stewardship interventions should look to reduce mortality as a measure of impact. More commonly, it is likely to be used as a balancing measure, assuring stakeholders that an ASP intervention does not lead to increased harm. Indeed, limited studies have shown that increasing appropriate antimicrobial therapy can reduce mortality, but there are a sizable number of studies that show that efforts to reduce excessive prescribing do not result in excess mortality [62••].

To focus the relationship between antimicrobial use and mortality, organism-specific or syndrome-specific mortality can be used [65, 66]. For example, programs may look at efforts to improve management of CAP, and then look at CAP-related mortality, in addition to other metrics.

### Length of stay

Length of stay is often an easy to obtain metric. It suffers from many of the same problems as mortality, especially with secular trends in developed countries where an emphasis has been placed on early discharges. Attempts to discontinue or transition to oral antimicrobial therapy from parenteral therapy has, perhaps, the strongest relationship to length of stay [41, 67, 68••, 69]. Rather than hospital length of stay, some interventions use intensive care unit length of stay as a surrogate for clinical improvement [68••, 69].

### Cure

There are various ways to measure cure of an infectious disease. There is clinical cure (whereby the patient is believed to be clinically well after effective treatment of their infection), microbiological cure (whereby microbiological cultures or other tests demonstrate that the pathogen is no longer present in a manner capable of causing disease), and other forms (e.g., radiographic cure). These are potentially useful metrics, but are very difficult for programs to reliably measure on a consistent basis. Further, it is unclear if this adds much above and beyond mortality and/or length of stay.

## Conclusion

Antimicrobial stewardship is a young field, with much room for growth, and with tremendous opportunity to improve healthcare. However, as McGowan points out in his definitive 2011 review of the limitations of contemporary research in antimicrobial stewardship, the opportunities lie in focusing on outcomes [26••].

## References

[1] Dunagan WC, Woodward RS, Medoff G (1989). Antimicrobial misuse in patients with positive blood cultures. Am J Med.

[2] Fridkin SK, Steward CD, Edwards JR (1999). Surveillance of antimicrobial use and antimicrobial resistance in United States hospitals: project ICARE phase 2. Project Intensive Care Antimicrobial Resistance Epidemiology (ICARE) hospitals. Clin Infect Dis.

[3] Jenkins TC, Sabel AL, Sarcone EE, Price CS, Mehler PS, Burman WJ (2010). Skin and soft-tissue infections requiring hospitalization at an academic medical center: opportunities for antimicrobial stewardship. Clin Infect Dis.

[4] Vincent JL, Rello J, Marshall J (2009). International study of the prevalence and outcomes of infection in intensive care units. JAMA.

[5] Goossens H (2009). Antibiotic consumption and link to resistance. Clin Microbiol Infect.

[6] Malhotra-Kumar S, Lammens C, Coenen S, Van Herck K, Goossens H (2007). Effect of azithromycin and clarithromycin therapy on pharyngeal carriage of macrolide-resistant streptococci in healthy volunteers: a randomised, double-blind, placebo-controlled study. Lancet.

[7] Chen DK, McGeer A, de Azavedo JC, Low DE (1999). Decreased susceptibility of Streptococcus pneumoniae to fluoroquinolones in Canada. Canadian Bacterial Surveillance Network. N Engl J Med.

[8] Hopkins S (2013). Improving antimicrobial stewardship and surveillance: the Chennai Declaration. BMJ.

[9.••] Policy statement on antimicrobial stewardship by the Society for Healthcare Epidemiology of America (SHEA), the Infectious Diseases Society of America (IDSA), and the Pediatric Infectious Diseases Society (PIDS). Infect Control Hosp Epidemiol. 2012;33(4):322–27. The most recent consensus statement on antimicrobial stewardship by the three US infectious diseases societies with the most interest in antimicrobial stewardship and resistance. 10.1086/665010 22418625

[10] Dellit TH, Owens RC, McGowan JE (2007). Infectious Diseases Society of America and the Society for Healthcare Epidemiology of America guidelines for developing an institutional program to enhance antimicrobial stewardship. Clin Infect Dis.

[11] McGowan JE, Gerding DN (1996). Does antibiotic restriction prevent resistance?. New Horiz.

[12.••] Morris AM, Brener S, Dresser L (2012). Use of a structured panel process to define quality metrics for antimicrobial stewardship programs. Infect Control Hosp Epidemiol.

[13.•] Schirmer PL, Mercier RC, Ryono RA (2012). Comparative assessment of antimicrobial usage measures in the Department of Veterans Affairs. Infect Control Hosp Epidemiol.

[14] WHO Collaborating Centre for Drug Statistics Methodology (2012). Guidelines for ATC classification and DDD assignment, 2013.

[15] Zagorski BM, Trick WE, Schwartz DN (2002). The effect of renal dysfunction on antimicrobial use measurements. Clin Infect Dis.

[16] Polk RE, Fox C, Mahoney A, Letcavage J, MacDougall C (2007). Measurement of adult antibacterial drug use in 130 US hospitals: comparison of defined daily dose and days of therapy. Clin Infect Dis.

[17] MacDougall C, Polk RE (2005). Antimicrobial stewardship programs in health care systems. Clin Microbiol Rev.

[18] Kubin CJ, Jia H, Alba LR, Furuya YE (2012). Lack of significant variability among different methods for calculating antimicrobial days of therapy. Infect Control Hosp Epidemiol.

[19] Fagon JY, Chastre J, Wolff M (2000). Invasive and noninvasive strategies for management of suspected ventilator-associated pneumonia. A randomized trial. Ann Intern Med.

[20] Chastre J, Wolff M, Fagon JY (2003). Comparison of 8 vs 15 days of antibiotic therapy for ventilator-associated pneumonia in adults: a randomized trial. JAMA.

[21] Goff DA, Bauer KA, Reed EE, Stevenson KB, Taylor JJ, West JE (2012). Is the “low-hanging fruit” worth picking for antimicrobial stewardship programs?. Clin Infect Dis.

[22] Hayman JN, Sbravati EC (1985). Controlling cephalosporin and aminoglycoside costs through pharmacy and therapeutics committee restrictions. Am J Hosp Pharm.

[23] Pate PG, Storey DF, Baum DL (2012). Implementation of an antimicrobial stewardship program at a 60-bed long-term acute care hospital. Infect Control Hosp Epidemiol.

[24] Niwa T, Shinoda Y, Suzuki A (2012). Outcome measurement of extensive implementation of antimicrobial stewardship in patients receiving intravenous antibiotics in a Japanese university hospital. Int J Clin Pract.

[25] Micek S, Johnson MT, Reichley R, Kollef MH (2012). An institutional perspective on the impact of recent antibiotic exposure on length of stay and hospital costs for patients with gram-negative sepsis. BMC Infect Dis.

[26.••] McGowan JE (2012). Antimicrobial stewardship—the state of the art in 2011: focus on outcome and methods. Infect Control Hosp Epidemiol.

[27] Bantar C, Sartori B, Vesco E (2003). A hospitalwide intervention program to optimize the quality of antibiotic use: impact on prescribing practice, antibiotic consumption, cost savings, and bacterial resistance. Clin Infect Dis.

[28] Vincent JL, Bihari DJ, Suter PM (1995). The prevalence of nosocomial infection in intensive care units in Europe. Results of the European Prevalence of Infection in Intensive Care (EPIC) Study. EPIC International Advisory Committee. JAMA.

[29.•] Malcolm W, Nathwani D, Davey P (2013). From intermittent antibiotic point prevalence surveys to quality improvement: experience in Scottish hospitals. Antimicrob Resist Infect Control.

[30] Robert J, Pean Y, Varon E (2012). Point prevalence survey of antibiotic use in French hospitals in 2009. J Antimicrob Chemother.

[31] Ansari F, Erntell M, Goossens H, Davey P (2009). The European surveillance of antimicrobial consumption (ESAC) point-prevalence survey of antibacterial use in 20 European hospitals in 2006. Clin Infect Dis.

[32.••] Kwint HM, van der Linden PD, Roukens MM, Natsch S (2012). Intensification of antibiotic use within acute care hospitals in the Netherlands. J Antimicrob Chemother.

[33] Avdic E, Cushinotto LA, Hughes AH (2012). Impact of an antimicrobial stewardship intervention on shortening the duration of therapy for community-acquired pneumonia. Clin Infect Dis.

[34] Hayashi Y, Paterson DL (2011). Strategies for reduction in duration of antibiotic use in hospitalized patients. Clin Infect Dis.

[35] Hochreiter M, Kohler T, Schweiger AM (2009). Procalcitonin to guide duration of antibiotic therapy in intensive care patients: a randomized prospective controlled trial. Crit Care.

[36] Hedrick TL, Evans HL, Smith RL (2006). Can we define the ideal duration of antibiotic therapy?. Surg Infect (Larchmt).

[37] Boyce JM, Pop OF, Abreu-Lanfranco O (2013). A trial of discontinuation of empiric vancomycin therapy in patients with suspected methicillin-resistant Staphylococcus aureus healthcare-associated pneumonia. Antimicrob Agents Chemother.

[38] Bolon MK, Arnold AD, Feldman HA, Goldmann DA, Wright SB (2005). An antibiotic order form intervention does not improve or reduce vancomycin use. Pediatr Infect Dis J.

[39] Sharma PR, Barman P (2010). Antimicrobial consumption and impact of “reserve antibiotic indent form” in an intensive care unit. Indian J Pharmacol.

[40] Nicolau DP, Carmeli Y, Crank CW (2012). Carbapenem stewardship: does ertapenem affect Pseudomonas susceptibility to other carbapenems? A review of the evidence. Int J Antimicrob Agents.

[41] Buising KL, Thursky KA, Robertson MB (2008). Electronic antibiotic stewardship—reduced consumption of broad-spectrum antibiotics using a computerized antimicrobial approval system in a hospital setting. J Antimicrob Chemother.

[42] Aldeyab MA, Kearney MP, Scott MG (2012). An evaluation of the impact of antibiotic stewardship on reducing the use of high-risk antibiotics and its effect on the incidence of Clostridium difficile infection in hospital settings. J Antimicrob Chemother.

[43] Talpaert MJ, Gopal Rao G, Cooper BS, Wade P (2011). Impact of guidelines and enhanced antibiotic stewardship on reducing broad-spectrum antibiotic usage and its effect on incidence of Clostridium difficile infection. J Antimicrob Chemother.

[44] Toth NR, Chambers RM, Davis SL (2010). Implementation of a care bundle for antimicrobial stewardship. Am J Health Syst Pharm.

[45] Elligsen M, Walker SA, Pinto R (2012). Audit and feedback to reduce broad-spectrum antibiotic use among intensive care unit patients: a controlled interrupted time series analysis. Infect Control Hosp Epidemiol.

[46] Cheng VC, To KK, Li IW (2009). Antimicrobial stewardship program directed at broad-spectrum intravenous antibiotics prescription in a tertiary hospital. Eur J Clin Microbiol Infect Dis.

[47] Burke JP (1998). Antibiotic resistance—squeezing the balloon?. JAMA.

[48] McCabe C, Kirchner C, Zhang H, Daley J, Fisman DN (2009). Guideline-concordant therapy and reduced mortality and length of stay in adults with community-acquired pneumonia: playing by the rules. Arch Intern Med.

[49] Richards MJ, Robertson MB, Dartnell JG (2003). Impact of a web-based antimicrobial approval system on broad-spectrum cephalosporin use at a teaching hospital. Med J Aust.

[50] Lee DH, Vielemeyer O (2011). Analysis of overall level of evidence behind Infectious Diseases Society of America practice guidelines. Arch Intern Med.

[51] Khan AR, Khan S, Zimmerman V, Baddour LM, Tleyjeh IM (2010). Quality and strength of evidence of the Infectious Diseases Society of America clinical practice guidelines. Clin Infect Dis.

[52] LaRosa LA, Fishman NO, Lautenbach E, Koppel RJ, Morales KH, Linkin DR (2007). Evaluation of antimicrobial therapy orders circumventing an antimicrobial stewardship program: investigating the strategy of “stealth dosing”. Infect Control Hosp Epidemiol.

[53] Jevons MP, Coe AW, Parker MT (1963). Methicillin resistance in staphylococci. Lancet.

[54] Zetola N, Francis JS, Nuermberger EL, Bishai WR (2005). Community-acquired meticillin-resistant Staphylococcus aureus: an emerging threat. Lancet Infect Dis.

[55] Climo MW, Israel DS, Wong ES, Williams D, Coudron P, Markowitz SM (1998). Hospital-wide restriction of clindamycin: effect on the incidence of Clostridium difficile-associated diarrhea and cost. Ann Intern Med.

[56] Ho M, Yang D, Wyle FA, Mulligan ME (1996). Increased incidence of Clostridium difficile-associated diarrhea following decreased restriction of antibiotic use. Clin Infect Dis.

[57] Kallen AJ, Thompson A, Ristaino P (2009). Complete restriction of fluoroquinolone use to control an outbreak of Clostridium difficile infection at a community hospital. Infect Control Hosp Epidemiol.

[58] Price J, Cheek E, Lippett S (2010). Impact of an intervention to control Clostridium difficile infection on hospital- and community-onset disease; an interrupted time series analysis. Clin Microbiol Infect.

[59] Thomas C, Riley TV (2003). Restriction of third generation cephalosporin use reduces the incidence of Clostridium difficile-associated diarrhoea in hospitalised patients. Commun Dis Intell.

[60] Valiquette L, Cossette B, Garant MP, Diab H, Pepin J (2007). Impact of a reduction in the use of high-risk antibiotics on the course of an epidemic of Clostridium difficile-associated disease caused by the hypervirulent NAP1/027 strain. Clin Infect Dis.

[61] Fishman N. Antimicrobial stewardship. Am J Infect Control. 2006;34(5 Suppl 1):S55–63; discussion S64–73. 10.1016/j.ajic.2006.05.237 16813983

[62.••] Davey P, Brown E, Charani E (2013). Interventions to improve antibiotic prescribing practices for hospital inpatients. Cochrane Database Syst Rev.

[63.•] Schulz LT, Fox BC, Polk RE (2012). Can the antibiogram be used to assess microbiologic outcomes after antimicrobial stewardship interventions? A critical review of the literature. Pharmacotherapy.

[64] Laxminarayan R, Klugman KP (2011). Communicating trends in resistance using a drug resistance index. BMJ Open.

[65] Slama TG (2008). Gram-negative antibiotic resistance: there is a price to pay. Crit Care.

[66] Ho PL, Cheng JCF, Ching PTY (2006). Optimising antimicrobial prescription in hospitals by introducting an antimicrobial stewardship programme in Hong Kong: consensus statement. Hong Kong Med J.

[67] Perez KK, Olsen RJ, Musick WL (2013). Integrating rapid pathogen identification and antimicrobial stewardship significantly decreases hospital costs. Arch Pathol Lab Med.

[68.••] Kaki R, Elligsen M, Walker S, Simor A, Palmay L, Daneman N (2011). Impact of antimicrobial stewardship in critical care: a systematic review. J Antimicrob Chemother.

[69] Claridge JA, Pang P, Leukhardt WH, Golob JF, Carter JW, Fadlalla AM (2010). Critical analysis of empiric antibiotic utilization: establishing benchmarks. Surg Infect (Larchmt).

